# A Multi-Stage Approach to Breast Cancer Classification Using Histopathology Images

**DOI:** 10.3390/diagnostics13010126

**Published:** 2022-12-30

**Authors:** Arnab Bagchi, Payel Pramanik, Ram Sarkar

**Affiliations:** Department of Computer Science and Engineering, Jadavpur University, Kolkata 700032, West Bengal, India

**Keywords:** breast cancer, histopathology images, deep learning, BACH dataset, ensemble learning

## Abstract

Breast cancer is one of the deadliest diseases worldwide among women. Early diagnosis and proper treatment can save many lives. Breast image analysis is a popular method for detecting breast cancer. Computer-aided diagnosis of breast images helps radiologists do the task more efficiently and appropriately. Histopathological image analysis is an important diagnostic method for breast cancer, which is basically microscopic imaging of breast tissue. In this work, we developed a deep learning-based method to classify breast cancer using histopathological images. We propose a patch-classification model to classify the image patches, where we divide the images into patches and pre-process these patches with stain normalization, regularization, and augmentation methods. We use machine-learning-based classifiers and ensembling methods to classify the image patches into four categories: normal, benign, in situ, and invasive. Next, we use the patch information from this model to classify the images into two classes (cancerous and non-cancerous) and four other classes (normal, benign, in situ, and invasive). We introduce a model to utilize the 2-class classification probabilities and classify the images into a 4-class classification. The proposed method yields promising results and achieves a classification accuracy of 97.50% for 4-class image classification and 98.6% for 2-class image classification on the ICIAR BACH dataset.

## 1. Introduction

Breast cancer is one of the most dangerous and fatal diseases in women around the world. Breast cancer has been ranked the number one cancer among Indian females. It has an age-adjusted rate as high as 25.8 per 100,000 women and a mortality rate of 12.7 per 100,000 women. The breast cancer projection for India in 2020 suggests the number will go as high as 1,797,900. Better health awareness and availability of breast cancer screening programs and treatment facilities would cause a favorable and positive clinical picture in the country [1]. According to the American Cancer Society (ACS), breast cancer is the most common cancer found in American women, except for skin cancers. The average risk of a woman in the U.S. developing breast cancer sometime in her life is about 12% or a 1 in 8 chance. The chance that a woman will die from breast cancer is about 2.6% or a 1 in 38 chance. According to a 2013 World Health Organisation (WHO) report, in 2011, around half a million women worldwide succumbed to breast cancer. The mortality rate of breast cancer is very high compared to other types of cancer [2]. The prognosis for cancer patients varies depending on the type of cancer. Patients with small-cell lung carcinoma have the worst prognosis. Surgery, chemotherapy, radiation, targeted therapy, and immunotherapy are currently used to treat cancer. These methods of curative treatment are only available to people with localized disease or treatment-sensitive malignancies; therefore, their contributions to cancer curability are limited [3]. However, early diagnosis significantly increases treatment success. Specifically, during the diagnosis procedure, specialists evaluate both overall and local tissue organization via whole-slide and microscopy images. However, the large amount of data and complexity of the images make this task time-consuming and non-trivial. As a result, the development of automatic detection and diagnosis tools is both difficult and necessary in this field. Breast cancer diagnosis consists of a screening test such as mammography or ultrasound whenever a lump is detected, followed by a biopsy and histopathological examination to make a definite diagnosis of any malignant growth in the breast tissue. The tissues can either be normal, benign, or malignant. Malignant lesions can be classified as either “in situ,” where the cells are restrained inside the mammary ductal-lobular system, or “invasive,” where the cells spread beyond that structure.

Breast-cancer prediction is usually performed by image analysis. Radiologists use different types of imaging techniques to predict the severity of breast cancer, which include diagnostic mammography, thermography, ultrasound, histology, or magnetic resonance imaging (MRI).

A mammography test is called a mammogram, which helps detect and diagnose breast cancer in women early [4]. It involves small doses of X-rays to produce a breast image [5]. Mammography is the cornerstone of population-level breast cancer screening and can detect more in situ lesions and smaller invasive cancers than other screening approaches, such as MRI and ultrasound [6]. Ultrasound uses a small range of frequencies to create images of the breast but keeps the contrast very small. Ultrasound can detect and identify breast mass nodes and is mostly used due to its ease, volume, non-invasiveness, and low cost [7].

MRI uses magnetic fields to construct very accurate 3-dimensional (3D) transverse images. Human body MRI requires a high dose of radiation to get accurate breast 3D images. Hence, the differences in the infected region are very vivid when we use an MRI and thus reveal cancer that cannot be seen in any other way. However, the problem is that MRI is much more costly compared to other cancer detection techniques [8].

Thermography is an effective screening test that can detect breast cancer by showing the body parts with irregular temperature shifts in a thermal image. Thermal images are captured using a thermal infrared camera that captures the infrared radiation emitted by the breast region and transforms this infrared radiation into electrical signals [8].

Histopathological images are microscopic images of the tissues used in disease analysis. The nature of histopathological images, therefore, makes this job lengthy, and the findings may be subject to the pathologist’s subjectivity. Therefore, the production of automated and precise methods of histopathological image analysis is a crucial area of research [9], and computer-aided analysis of histopathological images plays a significant role in the diagnosis of breast cancer and its prognosis [10,11]. However, the process of developing tools for performing this analysis is impeded by the following challenges. To be specific, histopathological images of breast cancer are fine-grained, high-resolution images that depict rich geometric structures and complex textures. The variability within a class and the consistency between classes can make classification extremely difficult, especially when dealing with multiple classes. Due to this, the extraction of discriminative features for histopathological images of breast cancer becomes a challenging task.

The state-of-the-art methods can be broadly categorized into two most common approaches for designing image-based recognition systems: (i) using visual feature descriptors or handcrafted features and (ii) DL-based methods using CNNs. The traditional approach involves using handcrafted features for segmentation of nuclei and cells from the breast-histology images to extract discernible features to distinguish between malignant and non-malignant tissues. These handcrafted features revolve around using active contours, thresholding, graph cuts, watershed segmentation, pixel-wise classification and clustering, or a combination of these. Rajathi in 2020 [12] used a radial basis neural network for classification purposes. The radial basis neural network was designed with the help of the optimization algorithm. The optimization tunes the classifier to reduce the error rate with the least amount of time spent on the training process. The cuckoo search algorithm was used for this purpose. Roy et al. [13] ensembled texture and statistical features using a stacking method. In this, redundant features were discarded using the Pearson’s correlation coefficient-based feature selection method. The majority of these methods concentrated on a two-class classification task of malignant or non-malignant tissues [14, 15]. For instance, Basavanhally et al. in 2011 [14] used the O’Callaghan neighborhood to solve the problem of tubule identification on hematoxylin and eosin (H & E)-stained breast cancer (BCa) histopathology images, where a tubule is characterized by a central lumen surrounded by cytoplasm and a ring of nuclei around the cytoplasm. The detection of tubules is important because tubular density is an important predictor in determining the grade of cancer. The potential lumen areas are segmented using a hierarchical normalized cut (HNCut) and an initialized color-gradient-based active contour model (CGAC). Evaluation of 1226 potential lumen areas from 14 patient studies produced an area under the receiver operating characteristic curve (AUC) of 0.91, along with the ability to classify true lumens with 86% accuracy. Dundar et al. in 2011 [15] evaluated digitized slides of tissues for certain cytological criteria and classified the tissues based on the quantitative features derived from the images. The system was trained using a total of 327 regions of interest (ROIs) collected across 62 patient cases and tested with a sequestered set of 149 ROIs collected from 33 patient cases. An overall accuracy of 87.9% was achieved on the test data. The test accuracy of 84.6% was obtained in the borderline case. Melekoodapattu et al. (2022) [16] implemented a system for autonomously diagnosing cancer using an integration method, which includes CNN and image texture attribute extraction. The nine-layer customized convolutional neural network is used to categorize data in the CNN stage. To improve the effectiveness of categorization in the extraction-based phase, texture features are defined and their dimensions are reduced using Uniform Manifold Approximation and Projection (UMAP). The findings of each phase were combined by an ensemble algorithm to arrive at the ultimate conclusion. The final categorization is presumed to be malignant if any of the stage’s output is malignant. In the MIAS repository, the ensemble method’s testing specificity and accuracy were 97.8% and 98%, respectively, whereas on the DDSM repository, they were 98.3% and 97.9%. However, these handcrafted features and engineering techniques suffered from some major drawbacks. This is because extracting high quality features from low resolution images is very difficult. Then comes another drawback of combining patch-level classification with image-level classification. Usually, patch-level classification is not very effective; to obtain good image-level accuracy, the patch features have to be used in a proper and efficient way.

Breast cancer is one of the few cancers for which an effective screening test is available. Breast cancer detection has been one of the most important fields in the computer vision field. Many DL-based networks have been used by researchers in the past [17,18,19,20,21,22,23]. There are basically two ways of making breast cancer predictions: using DL models and using feature descriptors. In current scenarios where computational capability is increasing exponentially, it is becoming quite natural to utilize it to the fullest to save both time and human effort. Moreover, since huge amounts of computational power are required for prediction using DL models, there is no scope for compromising with human efficiency. Moreover, machines, when trained, can gather vast amounts of information and use it to predict in a short amount of time.

For instance, in this paper [24], Melekoodappattu et al. proposed a model using extreme learning machines (ELM) with the fruitfly optimization algorithm (ELM-FOA) to tune the input weight to obtain optimal output at the ELM’s hidden node to obtain the solution analytically. The testing sensitivity and precision of ELM-FOA were 97.5% and 100%, respectively. The developed method can detect calcifications and tumors with 99.04% accuracy. Nirmala et al. in 2021 [25] proposed a new methodology for integrating the run-length features with the bat-optimized learning machine—BORN. BORN also features the most efficient visual saliency segmentation process to obtain a highly efficient diagnosis. BORN was tested with two different datasets, MIAS and DDSM, with different learning kernels, and compared to other intelligent algorithms, such as RF-ELM, EGAM, and associate classifiers. The proposed classifier achieved 99.5% accuracy. In [26], a soft computing classifier for the seven different CNN models was proposed for breast cancer histopathology-image classification. The proposed methodology uses the basic CNN with four convolutional layers, the basic CNN with five layers (with data augmentation), the VGG-19 transfer-learned model (with and without data augmentation), the VGG-16 transfer-learned model (without data augmentation), and the Xception transfer-learned model (with and without data augmentation). It uses seven models to extract features and then passes them through the classifier to make the final predictions. The dataset used in this research was the ICIAR BACH dataset for experimentation, and the model achieved a maximum accuracy of 96.91%. The primary drawback of this method is that training using the seven CNN models takes a huge amount of memory and time. Preetha et al. [27] proposed a PCA–LDA-based feature extraction and reduction (FER) technique that reduces the original feature space to a large extent. For classification, an ANNFIS classifier that uses the neural network concept with some fuzzy rule logic was used. In another paper [28], the authors combined two DCNNs to extract distinguished image features using the concept of transfer learning. The pre-trained Inception and Xceptions models were used in parallel. Then, the feature maps were combined and reduced by dropout before being fed to the last fully connected layer for classification. Then followed sub-image classification and whole-image classification based on a majority vote and maximum probability rules.

In this work, four tissue malignancy levels are considered: normal, benign, in situ carcinoma, and invasive carcinoma. The experiments were performed on the BACH dataset. The overall accuracy for the sub-image classification was 97.29%, and for the carcinoma cases, the sensitivity achieved was 99.58%. Wang et al. [29] proposed a hybrid model with a CNN (for feature extraction) and support vector machine (SVM) (for classification) to classify breast cancer histology images into four classes, benign, normal, in situ, and invasive, for the ICIAR BACH dataset. In addition to traditional image augmentation techniques, this study introduced deformation to microscopic images and then used a multi-model vote to obtain a validation accuracy of 92.5% and a test accuracy of 91.7%. This model used Xception and Inception ResNet v2 as the backbone models of the CNN. Nazeri et al. [30] proposed two consecutive CNNs to predict the classes of breast-cancer images. Firstly, due to the high resolution of the images, the images were converted to 512 × 512 resolution patches, and patch features were extracted by the first CNN. The second CNN utilized these patch features to obtain final image classification. It obtained 95% accuracy on the validation dataset. Training the patch-wise network with the same labels as the image-wise network is a disadvantage to the model’s performance because not every patch in an image represents the same category. Golatkar et al. [31] proposed a nuclei-based patch extraction strategy and fine-tuned a pretrained Inception-v3 network to classify the patches. It obtained an average accuracy of 85% for the four classes and 93% for non-cancer (i.e., normal or benign) vs. malignant (in situ or invasive carcinoma). The authors used majority voting in the patch predictions to classify the images. Sanyal et al. [32] used a patch classification ensemble technique for the ICIAR BACH Dataset 2018. At first, the patches are stain-normalized and then passed through VGG-19, Inception-ResNet v2, Inception v3, and ResNet101, and the posterior probabilities are then classified using the XGBoost classifier. Then, all five classifications (four from the models and one from the classifier) are ensembled to obtain the patch-prediction probabilities. After that, in the image-wise pipeline, the patch probabilities were ensembled using mean, product, weighted mean, weighted product, and majority voting to obtain the final image classification. This model obtained a 4-class classification accuracy of 95% and a 2-class classification accuracy of 98.75%.

To capture more discriminant deep features for pathological breast-cancer images, Zou et al. [33] introduced a novel attention high-order deep network (AHoNet) by simultaneously embedding attention mechanisms and high-order statistical representation into a residual convolutional network. AHoNet first employed an efficient channel attention module with non-dimensionality reduction and local cross-channel interaction to achieve local salient deep features of pathological breast-cancer images. Then, their second-order covariance statistics were further estimated through matrix power normalization, which provides a more robust global feature presentation of pathological breast-cancer images. AHoNet achieved optimal patient-level classification accuracy of 85% on the BACH database. Vang et al. [34] fine-tuned a pre-trained Inception-v3 network on the extracted patches. To obtain image-wise prediction, the patch-wise prediction was ensembled using majority voting, logistic regression, and gradient boosting trees. Mohamed et al. [35] proposed a deep learning approach to detect breast cancer from biopsy microscopy images, in which they examined the effects of different data preprocessing techniques on the performances of deep learning models. They introduced an ensemble method for aggregating the best models in order to improve performance. They showed that Densenet 169, Resnet 50, and Resnet 101 were the three best models, achieving accuracy scores of 62%, 68%, and 85%, respectively, without data pre-processing. With the help of data augmentation and segmentation, the accuracy of these models increased by 20%, 17%, and 6%, respectively. Additionally, the ensemble learning technique improved the accuracy of the models even further. The results show that the best accuracy achieved was 92.5%.

Many methods have been proposed to classify histology images for the ICIAR BACH 2018 dataset, which is an extension of the Bio-imaging 2015 dataset. In all these papers [12–16, 24–27], the high-resolution histology images (1536 × 2048) were pre-processed using different techniques and then segmented into patches. The patches were then passed through fine-tuned, pre-trained deep learning models such as VGG-19, Inception, Xception, and ResNet, and the patch-level results were obtained. After that, the patch-level results were used to obtain image-level results using different functions specific to each paper. Awan et al. [36] fine-tuned the ResNet model for patch-wise classification. Then, the trained ResNet model was used to extract deep feature representations from the patches. After that, the flattened features of 2 × 2 overlapping blocks of patches were used to train an SVM classifier, followed by a majority voting scheme for image-wise classification. Rakhlin et al. [37] proposed a transfer learning approach without fine-tuning based on extracting deep convolutional features from pre-trained networks. The authors used pre-trained CNNs to encode the patches to obtain sparse feature descriptors of low dimensionality, which were trained using a LightGBM classifier to classify the histopathology images.

With the advent of DL models, researchers have started to use these techniques for histopathological breast image classification and have achieved state-of-the-art results. DL is a subcategory of machine learning but is more efficient than machine learning classifiers due to its ability to learn new functions, unlike them, where there is a predefined classifier function. In this study, we used deep CNN models and multiple classification algorithms to form an ensemble for histopathology image classification. The main problem with this sort of learning algorithm is that high-resolution images take a huge amount of time to learn. Moreover, such images are difficult to train because different parts of the image require different attention weights. Hence, a viable alternative is to use learning from patches of the whole image and then ensemble it for the final image prediction.

In the present work, we propose a DL-based method to classify high-resolution breast histology images into one of the four classes: normal, benign, in situ, and invasive. We consider the images and divide them into training, testing, and validation sets. Then, we perform patch-level classification. The issue here is that we do not know what the patch labels are. What we know are the image labels. Thus, at the beginning, we assign each patch the same label as that of its parent image and design a model that outputs the probability of how likely it is that that patch label will be the same as that of the parent image. After that, we extract the deep features using a DL model. Lastly, we classify the patch as either normal, benign, in situ, or invasive using different classifiers and ensemble them. The final step is to convert the patch-level classification into an image-level classification. We take each image and create an array containing the number of patches corresponding to the four classes predicted by the patch prediction model for that image. Then, we pass this frequency array through a two-stage model to obtain the final prediction of four classes.

The key points of this work are as follows:

- We aimed to increase the image-level breast histopathology classification accuracy using a patch-level classification.
- We designed an efficient patch-level training model that is computationally fast. We fine-tuned a pre-trained model (pre-trained on the ImageNet dataset) with convolution, max pooling, and dense layers at the end. We obtained the patch features from this model.
- We introduce a two-stage model using a neural network to map the patch-level information to image-level prediction into two classes (cancerous and non-cancerous) and four classes (normal, benign, in situ, and invasive).
- We evaluated our model on a publicly available BACH histopathological dataset and achieved state-of-the-art classification accuracy of 97.50% for four classes and 98.6% for two classes.

The remainder of the paper is structured as follows. The proposed method is described in Section 2. The experimental results and analysis of the proposed method are presented in Section 3, where the discussion of the results is also presented. Finally, we conclude our work and state the limitations and some future possibilities in Section 4.

## 2. Materials and Methods

In this section, we first detail the dataset used for the experimentation, and then we discuss the complete process of our proposed model and go over each step in detail.

### 2.1. Dataset Description

The dataset used in this work was ICIAR 2018 BACH (BreAst Cancer Histology images) [38]. The dataset is composed of hematoxylin and eosin (H&E)-stained breast histology microscopy and whole-slide images. Microscopy images are labelled as normal, benign, in situ carcinoma, or invasive carcinoma according to the predominant cancer type or its absence in each image. The annotation was performed by two medical experts, and images where there was a disagreement were discarded. The dataset contains a total of 400 microscopy images; each of the four classes has 100 images. Microscopy images are in .tiff format and have the following specifications:

- Color model: RGB;
- Size: 1536 × 2048 pixels;
- Pixel scale: 0.42 µm × 0.42 µm;
- Memory space: 10–20 MB (approx.);
- Type of label: image-wise.

### 2.2. Methodology

**Figure 1:**
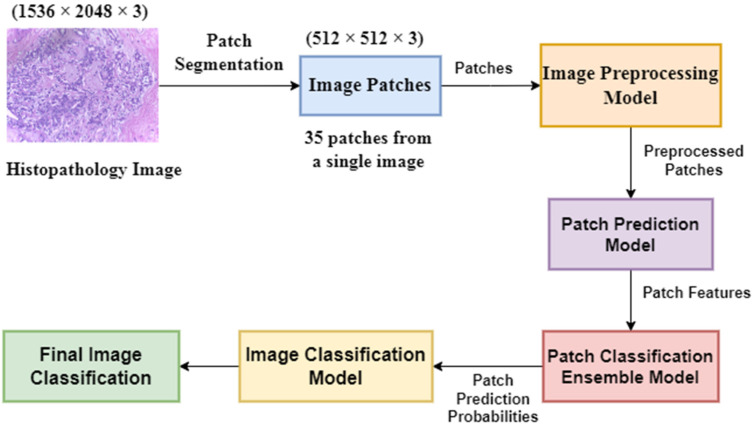
The overall pipeline of the proposed method.

First, we divided the dataset into training, testing, and valid sets. First, we split the 400 histology images into 80% as the intermediate set and 20% as the test set. Further, we divided the intermediate set into 65% for the training set and 15% for the validation set. In the later part of the whole model, after patch classification, we added the 15% of the validation data into the training data during image classification. Next, we divided the training and validation images into patches and trained a patch-level classifier. After that, we applied an image pre-processing model and normalized the images using the Macenko stain normalization [39] method. Then, we fed the images into different pre-trained deep learning models to extract model features and concatenated these features. Next, we converted these patch-level predictions to create a frequency array and merge the validation data with the training data. Then, we passed the features through different classifiers and ensembled them using different techniques, analyzing which ensemble technique works best. After that, we devised a neural network to map these patch-level predictions to image-level predictions. State-of-the-art methods have achieved this patchy image transition through different ensembling techniques, including the mean rule, product rule, max rule, etc. We designed a deep learning model and then leave the work of learning an appropriate function for the model. We divide the model into 2 broad classes: cancerous (invasive and in situ) and non-cancerous (normal and benign). Then, we use this information to modify the frequency array. Another neural network was created to classify the final image into four categories: normal, benign, in situ, and invasive. We basically made a two-stage neural network. As a result, we did not have to experiment with various ensemble methods to convert this patch-level classification into image-level classification. The overall pipeline of the proposed method is shown in Figure 1. Concisely, the following are the major steps of our proposed method:

- Dividing the dataset into training, testing, and validation sets.
- Patch segmentation and labelling.
- Pre-processing the image using a stain normalization technique and an image augmentation model.
- Feature extraction from patches using concatenation of features from different DL models.
- Patch classification using an ensemble of machine learning classifiers.
- Image classification using the patch classification results, where a two-stage neural network has been applied.

#### 2.2.1. Image Splitting

**Figure 2:**
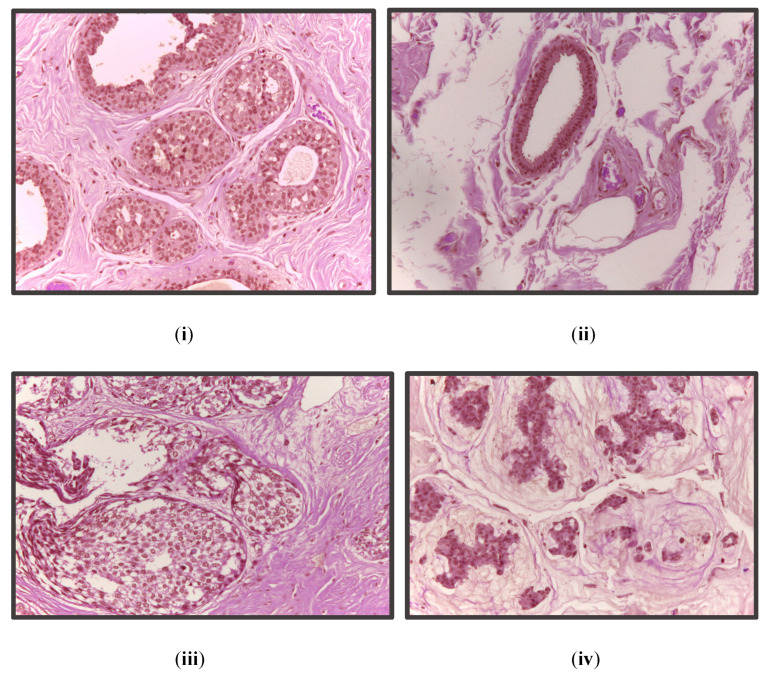
From top left in clockwise manner: (**i**) benign, (**ii**) normal, (**iii**) invasive, and (**iv**) in situ.

There are a total of 400 images with 1536 × 2048 resolution in the ICIAR BACH 2018 dataset [38]. The pixel values of the images are in the range 0 to 255. Thus, to prevent the values from exploding, we converted the pixel values into the range 0 to 1 by dividing by 255. Thus, for every image, we had 1536 × 2048 data points (pixels) with a standard deviation value of 1 and a mean value between 0 and 1. We used 65% of the dataset for the training, 15% of the dataset for validation, and the remaining 20% for the testing of the model. We added 15% of the validation data as training data in the latter part of this pipeline during image classification. We did this so that the image classification model can be trained using data from the validation set, resulting in a more robust model that performed better on the test set. Figure 2 shows all four classes of images in the dataset.

#### 2.2.2. Patch Segmentation and Labelling

Since these individual images have very high resolution, we converted the images into patches. The main problems with learning from high-resolution images are the time and space requirements. It is almost impossible to fit those high-resolution images into a model unless we have high-end resources. Thus, we divide each image into patches and then feed it to a model for learning. We consider the image and convert it into patches of 512 × 512 × 3 with an overlap of 50% with the adjacent patch to keep the spatial orientation of the data. The size of the patches is taken as 512 because making it smaller would mean the patches would get a much-magnified portion and may be misclassified by a learning model. Smaller patches, on the other hand, would fail to take the context of the images into account and would appear similar for different classes of images. Again, another problem is that it is not completely correct to label all 35 patches with the same label as the image of which they are part. The reason is that there are normal, healthy cells among the cancer cells in the invasive and in situ cases. As a result, some patches are less likely to have the same label as the image of which they are a part. For example, a cancerous cell image may have a patch that is predominantly healthy, i.e., normal or benign. Thus, the problem now is the patch labelling. Since we do not have a region of interest (ROI) for each cell, we assign the same label to the patch as to the image. Thus, all 35 patches (with a patch size of 512 × 512 and a stride of 256) from an image will have the same labels. L(i, j) represents the label of the jth patch of the ith image, which we assign as the ground truth. If the ground truth label of each image is represented as L(i), then l(i, j) can be defined as l(i, j) = L(i) for every j such that 1 ≤ j ≤ 35.

#### 2.2.3. Image Augmentation and Pre-Processing

Data normalization and regularization are the two most important aspects of any machine learning algorithm. Data normalization helps us overcome many training issues, such as vanishing or exploding gradients, faster training, and smoother descent to minima for the cost function. Data regularization, on the other hand, prevents the training data from over-fitting.

**Figure 3:**
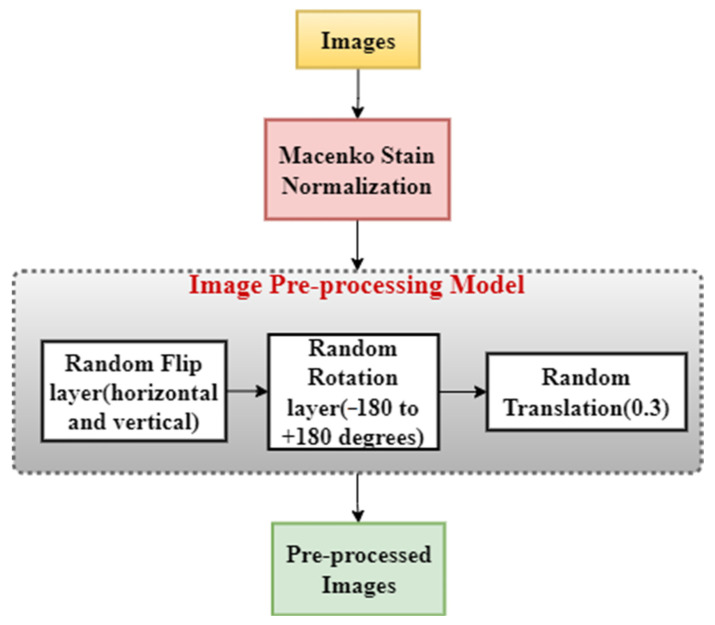
Image pre-processing method that consists of three sequential layers that take images as the input and give pre-processed images as the output.

We designed a sequential model, as shown in Figure 3, which adds regularized versions of the images to the train dataset. The model is:

- Horizontal and vertical flips randomly;
- Rotates by a random angle given as a parameter;
- Translates by a random fraction.

**Figure 4:**
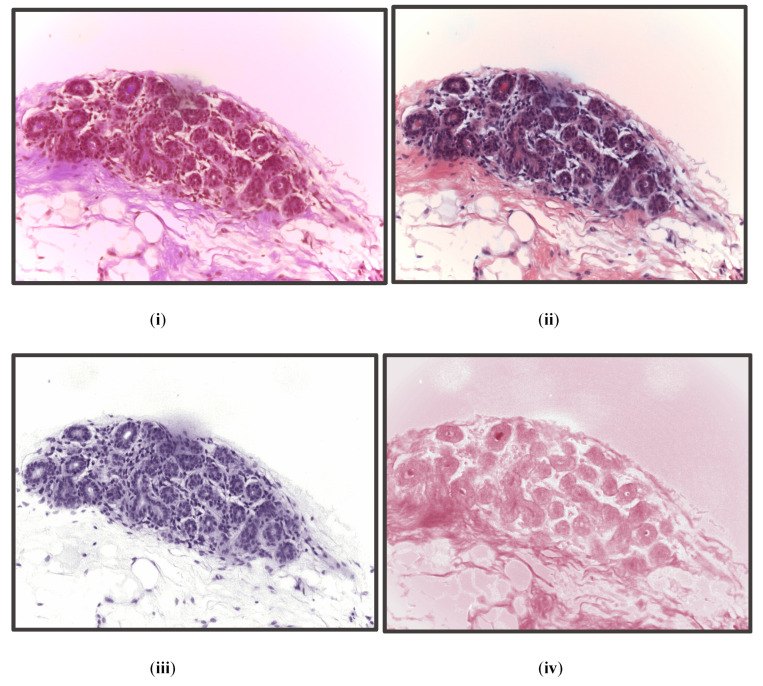
From top left in a clockwise direction: (**i**) original image, (**ii**) stain normalized image (**iii**) hematoxylin stain, and (**iv**) eosin stain.

At first, we pass the patches through Macenko stain normalization [39]. This basically converts all the images into the same fixed color space as the template image, which is taken to be one of the patches of the training dataset. This helps to give a constant base and reduce stain varieties across the dataset. This also gives a generic base for the images and helps increase the accuracy of the model. The original image, the stained normalized image, and the separated hematoxylin and eosin stain are all shown in Figure 4. The normalized image has a more contrasting effect that is even easier for the human eye to recognize than that of the original image. These random layers help to increase variety in the training dataset and help in generalizing the training dataset, which ultimately increases the size and orientation of training data and helps the model learn a greater variety of images. This increases the overall accuracy of the patch prediction model and prevents the model from over-fitting the training data.

#### 2.2.4. Patch-Level Feature Extraction

We designed a DL-based model to extract features from patches, with four outputs each representing the probability with which the patch belongs to that particular class, as mentioned earlier. Let “P” be the output vector of this model. P(i, j) is the probability with which the “ith” patch belongs to the “jth” class, where 1 ≤ j ≤ 4, which indicates each of the 4 classes: normal, benign, in situ, and invasive. We obtained the probability value from a softmax function applied at the last layer of the neural network.

We divided the model into two stages: first, dimension reduction, and second, fine-tuning. We designed a dimension reduction method using a pre-trained DL model. We first pass all the patches through a pre-trained model and use their trained weights to output a low-dimensional feature vector. We first fine-tuned a few deep learning models, VGG-19 [40], VGG-16 [40], Inception-ResNet v2 [41], Xception [42], and ResNet 50 [43]; and calculated the validation accuracy of these models. We found out that VGG-19, VGG-16, and Inception-ResNet v2 produce better accuracy than Xception and ResNet 50. Thus, we moved forward by taking VGG-19, VGG-16, and Inception-ResNet v2 as the dimension-reduction models.

**Figure 5:**
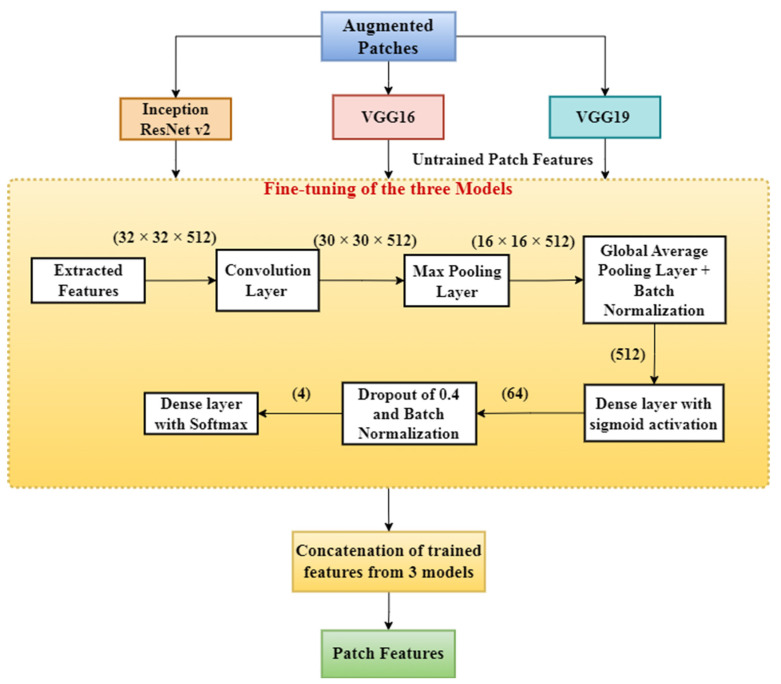
Feature extraction model that takes patches as the input and trains the model to provide output as a concatenated patch features set.

However, the problem is that these features are untrained and may not be completely suitable for this classification. Now comes the second stage, i.e., fine-tuning. Thus, we trained these features using a few convolutional, dropout, activation, batch–normalization and dense layers. The output was a dense layer of four nodes with softmax activation, as shown in Figure 5. We used a dropout of 0.4 and 64 neurons in the added layer. We also used L2 regularization, “He-uniform” as the kernel initializer and sigmoid as the activation function. The sigmoid function can be defined as follows:

(1) $$Sigmoid\;Function\;\left(x\right)=\frac{1}{\left(1+e^{\left(-x\right)}\right)}$$

Figure 5 depicts the extraction of the first untrained patches from the three models: VGG-19, VGG-16, and Inception-ResNet v2. The fine-tuning model was then used to train these features one by one to extract them. After that, the features were concatenated from the three separate models. In the fine-tuning model, the first layer had 512 kernels of size (3 × 3) with a stride of 1. The next max pooling layer had a (2 × 2) kernel and a stride of (2, 2). Then, the data passed through a GAP (global average pooling) layer, which resulted in the average values for every channel. Thus, for every channel, there was only one value left. Thus, the dimension became (batch_size x no. of channels). This then passed through the batch normalization layer, followed by two dense layers with 64 and 4 neurons, respectively. The first dense layer had a sigmoid activation function. Then, lastly, the data passed through a softmax activation function. Then, after the training of the model, we obtain the features from the output of the GAP layer. Thus, the features have 512 dimensions. Details of hyper-parameter tuning for the VGG19 model were as follows:

- A dropout value of 0.4 produced the best results.
- Learning rate: the learning rate was varied as lr = lr0/ (1 + decay rate) after each epoch. Lr0 was chosen as 0.008.
- Decay of learning rate: (lr0/number of epochs) is chosen.
- Momentum of the optimizer: any value between 0.5 and 0.9.
- Number of neurons in the dense layer: 64 neurons give the best result.

We compared the results of some pre-trained models for the purpose of dimension reduction and observed that the VGG-19 model gave the best result. During training, the models overfit after about 40 epochs, so it was necessary to stop early during the epochs when the validation accuracy increased by a negligible amount in the consecutive five epochs. Thus, we defined a callback function to do the same. We obtained the features from the second-to-last layer of the three pre-trained models (VGG-19, VGG-16, and Inception-ResNet v2) and then concatenated them. Since different models give different types of features, we allowed the overall model to learn as much as possible from all three models by concatenating the three feature sets from the three models. We concatenated the features after the fine-tuning stage, which were later classified by machine learning classifiers, as described in Section 2.2.5. We trained this model using the adam optimizer and the cross-entropy loss function. Cross-entropy loss is calculated according to Equation (2).

Cross Entropy Loss = −∑ y_original_ log y_predicted_ (2)

where y_original_ is the original label of the patch and y_predicted_ is the predicted label of the patch. Categorical accuracy is calculated as the ratio of the patches correctly predicted to the total number of patches.

(3) $$\mathrm{Categorical}\;\mathrm{Accuracy}=\frac{Patches\;Correctly\;Predicted}{Total\;Number\;of\;patches}$$

#### 2.2.5. Patch Classification

We obtained concatenated features from the feature extraction model, as shown in Figure 5. Now, to get output probabilities from the features, we need to classify these features into four different classes. In this stage, patch features are classified using different machine learning classifiers [44]. The classification algorithms used are:

- KNN.
- SVM.
- Random forest.
- Adaboost.
- XGB.

We took the patch features obtained in the previous step and then passed them through the different classifiers mentioned earlier. Different classifiers have different benefits, and no single classifier can provide all the information. Hence, to get combined information from different classification techniques based on the probability values obtained previously, we performed different ensemble techniques on the outputs of these classifiers, which are described in Section 2.2.6. Ensembling provided a higher level of accuracy than any of the individual standalone classifiers.

#### 2.2.6. Ensemble Techniques

**Figure 6:**
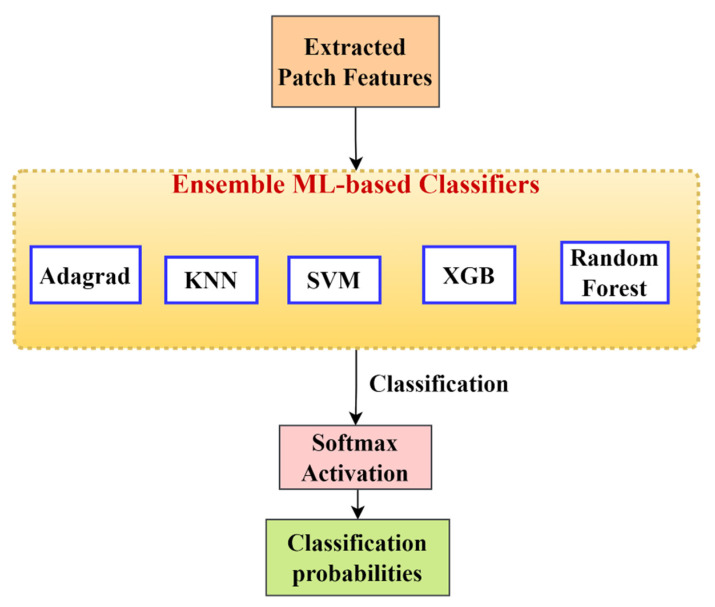
Ensemble technique that takes in extracted patch features and ensembles to give the classification probabilities for each patch.

An ensemble method is a machine learning technique that combines the outputs of several base models in order to produce one optimal predictive model. It basically uses different types of results, giving different types of information about an entity, and then combines them using an ensemble function to obtain more refined information. In this case, we use an ensemble technique, as shown in Figure 6. Different ensemble techniques are defined as follows:

- The average of probability values is taken for each patch across the five classifiers, i.e.,

Prob(i) = ∑Prob(i, j) for j = {KNN, SVM, Random Forest, Adaboost, XGB}(4)

- Multiplication technique:

Prob(i) = ΠProb(i, j) for j = {KNN, SVM, Random Forest, Adaboost, XGB}(5)

- Maximum technique:

Prob(i) = Max Prob(i, j) for j = {KNN, SVM, Random Forest, Adaboost, XGB}(6)

Out of these, we chose the one that produces the maximum accuracy as the final ensemble technique. Next, to normalize the probabilities, these one-hot probabilities were passed through a softmax activation layer.

#### 2.2.7. Image Classification: Part 1

**Figure 7:**
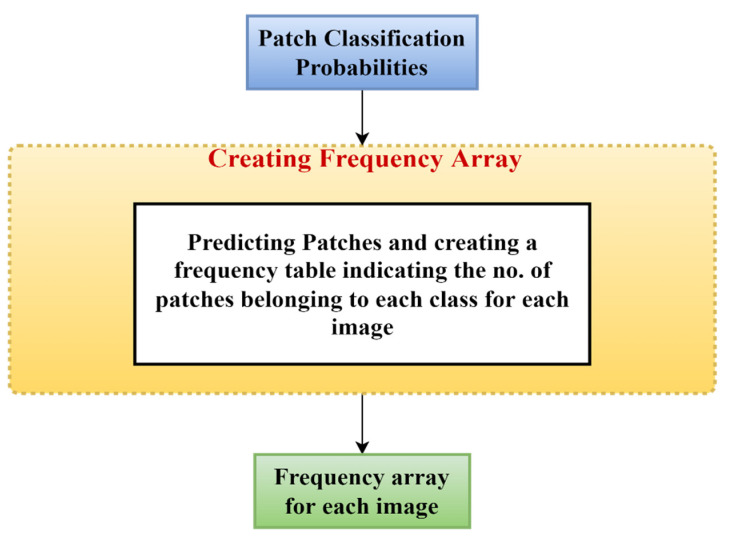
Creation of a frequency array for each image using the patch probabilities.

From patch feature extraction and patch classification we obtained the class of which a particular patch is a member. For each image, we generated a frequency array (as shown in Figure 7) that counts the numbers of normal, benign, in situ, and invasive patches. We also added the remaining 15% of the validation set for prediction with the original 65% train images at this stage. Then, we divided the patch predictions into 35 equal halves. As a result, the first 35 patches represent the first image, the following 35 patches represent the second image, and so on. Then, for each set of 35 patches, we defined a one-dimensional array of size 4. Array positions 0, 1, 2, and 3 represent the counts of normal, benign, in situ, and invasive patches of that image. Therefore, the array for the “ith” image would be Freq(i) = {Count (Normal), Count (Benign), Count (In situ), Count (Invasive)}, where Count(x) represents the frequency of patches predicted as type “x” of all the 35 patches belonging to “ith” image.

In this stage, we classify images into two classes: cancerous and non-cancerous. We consider both normal and benign types as non-cancerous, whereas in situ and invasive types as cancerous. For this, we created another frequency array, which can be defined as follows:

New_Freq(i) = [Cancerous Count, Non-Cancerous Count] for i = train images + validation images where, Non-cancerous Count = Normal Count + Benign Count and Cancerous Count = In situ Count + Invasive Count.

**Figure 8:**
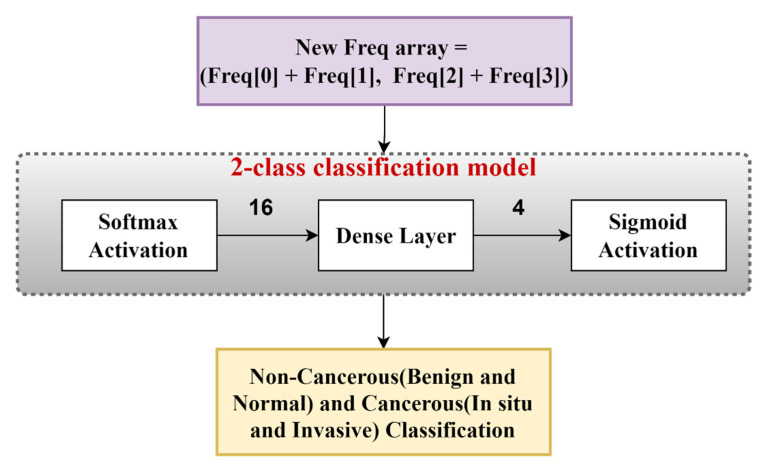
The 2-class image classification model that takes in the frequency array and uses it for the image classification into cancerous or non-cancerous images.

Now, to map the frequencies into patch predictions, we used a neural network to select combinations of these frequencies and a suitable activation for getting image-level predictions. The model shown in Figure 8 used the softmax activation layer, the dense layer with one output node with “L2” regularization, and the sigmoid activation. Here, we used the adam optimizer and the binary cross entropy loss function. We have also experimented with the RMSprop and the SGD as optimizer functions, but Adam produced the best result, as it combines the properties of both the RMSprop and the SGD.

From the 2-class classification model, we obtained a probability array that indicates whether the image is cancerous or non-cancerous. Then, we predicted the training images using the above method and considered the image to be cancerous if the probability was greater than 0.5, and non-cancerous otherwise. Let the sigmoid probability value for the “ith” image to be cancerous be P_i_. This means that the probability of the image being cancerous is P_i_, and the probability of it being non-cancerous is (1 − P_i_). Thus, the new modified frequency array will be defined as follows:

Freq(i) = [Benign Count X (1 − P_i_), Normal Count X (1 − P_i_), In situ Count X (P_i_), Invasive Count X (P_i_)] (7)

In this case, a factor of P_i_ is multiplied by the cancerous class (i.e., in situ and invasive types) and (1 − P_i_) by the non-cancerous class, resulting in a weighted frequency array that improves and accelerates learning. These probabilities are similar to attention weights that are used on different inputs. Thus, to make our input features, i.e., the frequency array, better, we used weights on them so that cancerous images are not predicted as non-cancerous or vice versa.

#### 2.2.8. Image Classification: Part 2

**Figure 9:**
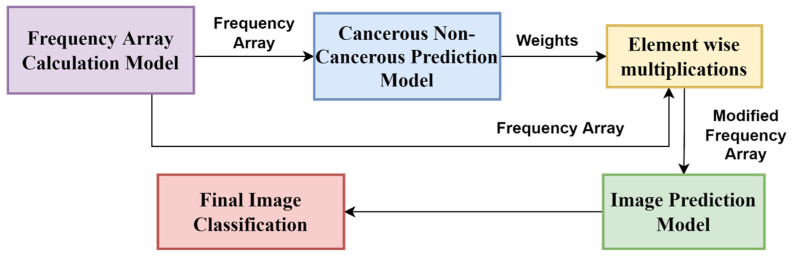
Image classification model using patch-prediction probabilities.

This stage helps in training the final image prediction model. By studying the old frequency array, it was found that cancerous images are rarely misjudged as non-cancerous or vice versa. Rather, it happens in intra-class scenarios. Normal cells are misjudged as benign, or vice versa. Similarly, in situ cells are misjudged as invasive, or vice versa. Thus, we classify the cells into cancerous and non-cancerous types, then perform multiplication (as shown in Figure 9) between the weights and the original frequency array to get the modified frequency array.

**Figure 10:**
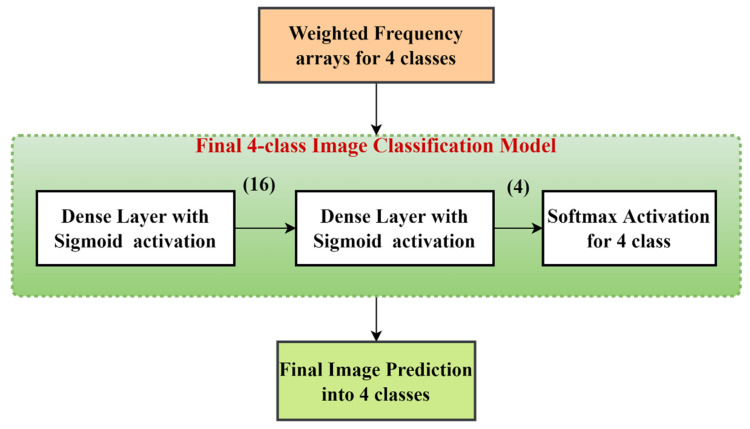
The 4-class classification model that takes the weighted frequency array for the images and trains the model to classify the images into one of the 4 classes as normal, benign, in situ, and invasive.

The modified frequency array is then subjected to a softmax activation to normalize the value. We pass these frequency arrays through our final image prediction model. The model depicted in Figure 10 employs the dense layer with four output neurons and sigmoid activation, and the dense layer with four output neurons and softmax activation. The final softmax layer yields the probabilities with which the image belongs to that class.

Output layer = {P1, P2, P3, P4}, where Pi is the probability with which the image belongs to the “ith” class for 1 ≤ i ≤ 4, where “i” represents either normal, benign, in situ, or invasive. During the training of this model, we provide a decay rate of approximately (learning rate/number of epochs) to the learning rate. The learning rate (lr) gets updated as lr = lr/ (1 + decay rate) after each epoch.

Performing convolution on high-resolution images is very time-consuming. Moreover, learning from a large image has its own problems, as the model sometimes fails to decide which parts of the image to emphasize and which parts to ignore. Thus, dividing images into patches is one of the ways to solve this problem. We trained the patches first, and then devised a method for mapping patch classification to image classification.

## 3. Results and Discussion

### 3.1. Experimental Setup

The computational system used for the work has a single GPU system P100 with 25 GB RAM and 128 GB storage. We used an IPython notebook for writing the code and used quite a few libraries to facilitate our model to get faster results. These libraries included tensorflow keras libraries for using the inbuilt layers such as Dense and Convolution, to name a few; matplotlib.pyplt for plotting the graphs of accuracy vs. epochs; sklearn for train–test-split function to divide the dataset into required sets.

### 3.2. Performance Evaluation Metrics

We evaluated our model using some standard performance metrics, based on which we can determine how well the model works for classifying the breast cancer histology images. We define the metrics as follows:

Accuracy is the measure of the ratio of correctly predicted labels to the total size of the dataset. It is defined by Equation (8).

(8) $$\mathrm{Accuracy}=\frac{True\;Positive+True\;Negative}{\left(True\;Positive+True\;Negative+False\;Positive+False\;Negative\right)}$$

Precision is defined as the proportion of correctly predicted labels in a class to the total number of samples in the class. It is calculated according to Equation (9).

(9) $$\mathrm{Precision}=\frac{True\;Positive}{\left(False\;Positive+True\;Positive\right)}$$

Recall is defined as the proportion of positive samples to all positive samples in that class.

(10) $$\mathrm{Recall}=\frac{True\;Positive}{\left(True\;Positive+False\;Negative\right)}$$

The F1-score is the weighted harmonic mean of the precision and recall scores.

(11) $$F1-\mathrm{score}=\mathrm{Harmonic}\;\mathrm{Mean}(\mathrm{Precision},\;\mathrm{Recall})=\frac{2\;*\;Precision\;*\;Recall}{\left(Precision+Recall\right)}$$

where true positive, true negative, false positive, and false negative are defined as follows:

True positive = number of images correctly classified as that class. 
False positive = number of images incorrectly classified as that class. 
False negative = number of images in that class incorrectly classified as some other class. 

### 3.3. Experimental Results

At first, we experimented with a few transfer learning models and analyzed the results obtained on the BACH dataset. Table 1 shows the accuracy of different fine-tuned models, which shows that small models such as VGG-16 and VGG-19 produce comparatively better results than the deeper models. For this experimentation, we froze all but the last layer for each model, then added a dense layer with softmax activation to get the class prediction. Next, the last two layers for each of the models were unfrozen, and we calculated the accuracy and went on increasing the number of unfrozen layers. The unfrozen layers were actually the ones that were being trained. We observed that after four layers of unfreezing, the accuracy was not increasing but rather experiencing a slight dip towards the end. This is because in the beginning, the amount of data was quite high for training small parameters, but as we went on unfreezing the layers, the number of parameters to train went on increasing, and an optimum was reached, after which the data became smaller in comparison to the number of parameters to train. Thus, we kept four unfrozen layers at the end while training the fine-tuned model. The number of epochs was generally 50–60, after which it started overfitting and there was a drop in validation accuracy. We used the adam optimizer with beta_1 set to 0.8, beta_2 set to 0.99, and the rest of the parameters left alone. Here, beta_1 and beta_2 are the decay rates of the first and second moment estimates, respectively. From Table 1, we can see that the fine-tuned VGG-16 model yielded the best result of 81.5% validation accuracy—superior to Inception-Resnet v2 and VGG-19. We see that lighter models such as VGG-16 or VGG-19 tend to give better results when huge amounts of data are present.

After fine-tuning the pre-trained model, we tried out different splitting combinations so as to find the optimal solution. The idea is to get enough data to train the parameters, but we also have to keep in mind not to overfit the model. Overfitting is one of the main problems in the training phase. To counteract this, we introduce the concept of a validation dataset. The validation set checks the cross-entropy loss and categorical accuracy after each epoch. This helps to check whether the accuracy of unseen data is increasing with epochs or the training data are being overfit. Hence, as soon as the validation accuracy reaches a flat area in the curve, we tend to stop the training at that point. From Table 2, we can see that the train–test split 80/20 worked best.

**Figure 11:**
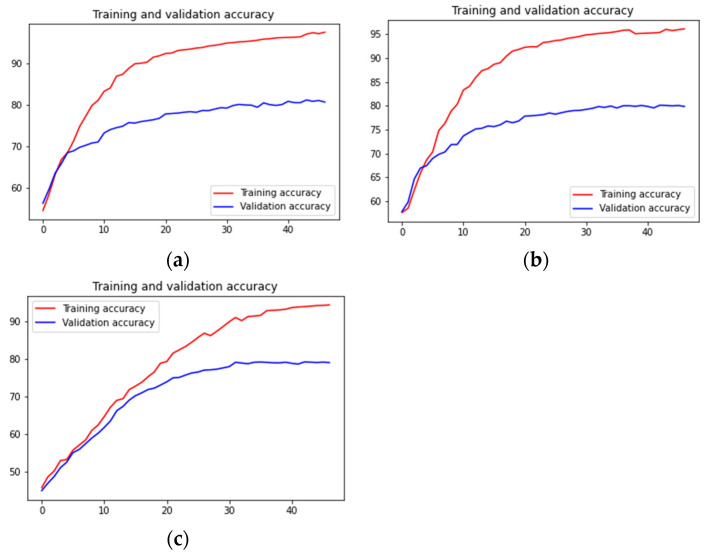
From top-left in a clockwise manner: training accuracy and validation accuracy vs. number of epochs of fine-tuned (**a**) VGG-16 model, (**b**) VGG-19 model, and (**c**) Inception-ResNet v2 model.

**Figure 12:**
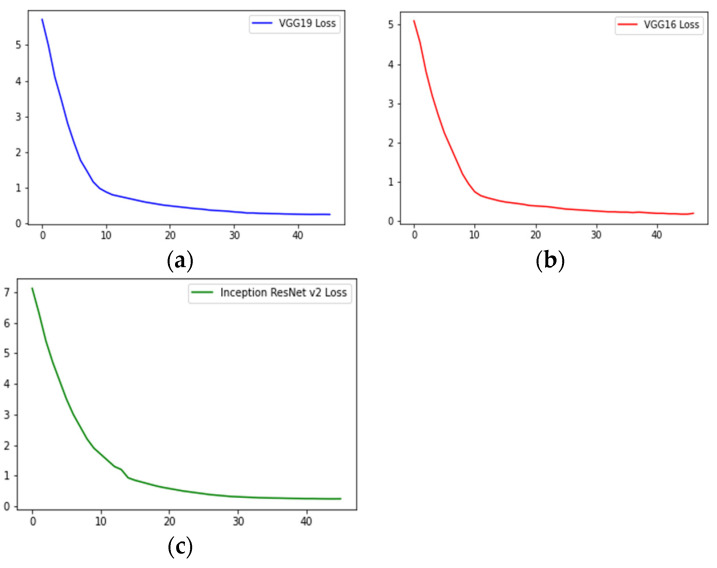
Validation loss vs. number of epochs of (**a**) VGG-16, (**b**) VGG-19, and (**c**) Inception-ResNet v2.

Figure 11 shows how the training and validation accuracies increased on increasing the number of epochs. It also shows the model overfits, but we stopped training when the validation accuracy did not increase when further increasing the number of epochs. Figure 12 shows the validation loss curves of the VGG-16, VGG-19 and Inception-ResNet v2 models.

#### 3.3.1. Patch Classification Results

A maximum accuracy of 81.5% was obtained with the VGG16 model, as shown in Table 1. After that, the features were concatenated, and the concatenated features were used for the classification using machine learning classifiers. The classification accuracy was listed in Table 3.

We extracted features from the three pre-trained models (VGG-19, VGG-16, and Inception-ResNet v2) and then fine-tuned them. After that, we concatenated the features. Now, after concatenating the features, we classified the features using machine learning classifiers, such as KNN, SVM, random forest, Adaboost, and XGBoost. Among the classifiers, SVM yielded the best result of 81.5% accuracy. We ensembled the classification results using the average, product, and maximum rules, as mentioned in Section 2.2.6. We obtained the final ensemble results, which used information from all the DCNN models and various ensemble techniques. Out of the three ensemble techniques, we found that the averaging technique produced the best accuracy of 82.5%. Since the ensemble uses all the information from all the classification techniques, it produces better results than any of them.

In Table 4, we can see the different performance metrics explained in Section 3.2 for all four classes of histopathology images. We found that invasive images had the highest precision and F1 score, whereas in situ images had the highest recall values.

#### 3.3.2. Image Classification Results

The model, as explained in Section 2.2.7, gives an accuracy of 98.6% in 2-class classification: benign (normal and benign) and malignant (invasive and in situ). However, going further into the model, we use the posterior probabilities only to modify the frequency array and use the probabilities as weights to amplify the input going into the final image classification model.

From Table 5, we can see that normal, in situ, and invasive have comparatively higher accuracy than benign images, and we achieved the highest overall accuracy of 97.50% across all the four classes, which is superior performance to many state-of-the-art models. Table 4 lists the performance metrics for the patches of the four classes. From Table 4, we can see that invasive patches have the highest precision, and in situ patches have the lowest precision. The reason is invasive images have very different structures compared to the other three classes. In situ images, on the other hand, have localized infections, and their infections seem as lobules in normal and benign cases. Additionally, another valid reason can be that while converting images to patches, we have labelled all the patches to have the same label as that of the image. Thus, it is difficult to even manually detect in situ images, so we achieved a low level of precision. However, in reality, we can have a few normal or benign patches in in situ images. Thus, labelling those patches as in situ will be like feeding wrong information to the model, which can have a negative impact on the accuracy of the patch prediction model.

**Figure 13:**
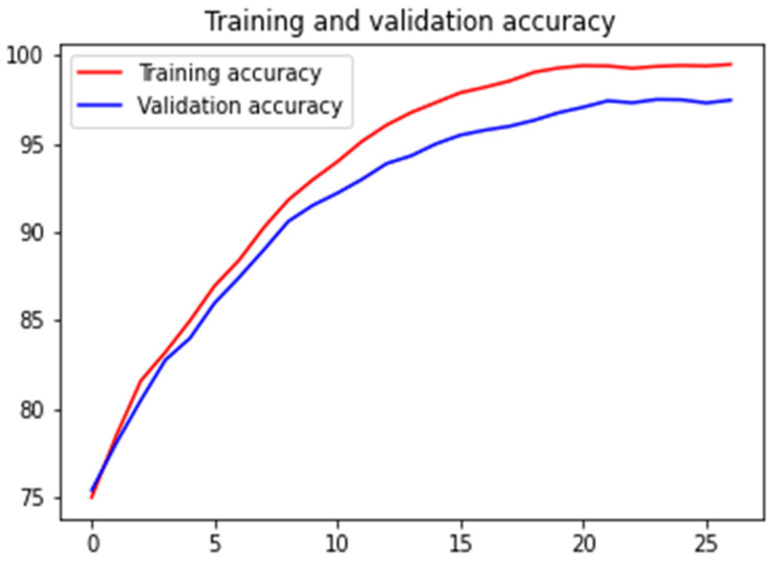
Training and validation accuracy vs. epoch for 4-class image classification.

After patch classification comes the part of developing a method that takes the patch-level output as input and uses it to get image-level output. For each image, as described in the proposed method section, we create a frequency array that indicates the number of patches that belong to each class for an image. Out of 35 patches for an image, something like {28, 4, 2, 1} indicates that 28 patches belong to class 1, 4 patches belong to class 2, 2 patches belong to class 3, and 1 patch belongs to class 4. Here, we added the 20% untrained validation data to this trained data to prevent the data from over-fitting. Now, we use a neural network that maps this frequency array to the final image prediction. The training and validation accuracies for 4-class image classification over the epochs are shown in Figure 13. An accuracy of 97.5% on the test dataset was obtained. In Table 5, we list different performance metrics, such as precision, recall, F1 score, and accuracy for the image-level classification.

We compared our proposed method with some of the modern methods and record the results in Table 6. It can be seen in Table 6 that our method outperforms the state-of-the-art methods considered here for comparison by a good margin.

## 4. Conclusions

Classification of breast-cancer tissues using histopathological images is a challenging task. In the present work, we proposed a DL-based method for breast cancer image classification with histopathological images. First, we introduced a model to classify image patches, as these images are of high resolution, and it is very expensive to deal with the images directly in DL-based models. We divide the images into patches, perform pre-processing, and classify these patches into four categories. After that, we use the information about patch probabilities from this model to convert this patch-level accuracy to image-level accuracy. In general, state-of-the-art models convert patch-level classification results to image-level predictions by using various ensembling techniques, such as averaging the results, taking the element wise product, or taking the maximum of all, but in this work, we designed a two-stage neural network to map the patch-level information to image-level prediction in four classes: normal, benign, invasive, and in situ. We compared the proposed method’s results with past methods, and it is shown that the method produces promising results and can be used as a reliable diagnostic tool for breast cancer classification from histopathological breast cancer images.

Our proposed model has achieved promising classification results with histopathological breast images. However, for this model, the major limitation is that we have to train quite a few models to achieve the ensemble accuracy, which is time-consuming and makes the entire model a little bit complex. Hence, development of an end-to-end deep learning model can be explored in future research attempts, where high-resolution images could be directly fed as input and hopefully produce good results. Additionally, the patch-level accuracy could be improved by designing a model that can assign patch-level labels more accurately, rather than manually assigning all the patches the same labels as the images of which they are parts. Other histopathological image datasets can be explored in the future for the robustness of the model.

## Figures and Tables

**Table 1:** Comparison of the fine-tuned pre-trained model accuracies of image patches. Bold font value indicates the highest value.

Model	Train Accuracy (%)	Validation Accuracy (%)
VGG16	97	**81.5**
Inception-resnet v2	96	79.5
VGG19	95	80
Xception	92	70
ResNet 50	93	72

**Table 2:** Comparing patch-level accuracies for different train–test splits. Bold font value indicates the highest value.

Train-Test Split	Train Accuracy (%)	Validation Accuracy (%)
60-40	96	78
70-30	98	79.5
75-25	99	80.5
80-20	99	**81.5**
90-10	99	81

**Table 3:** Training and validation accuracies of different classification algorithms on training of patch features. Bold font values indicate highest values.

Classification Algorithm	Training Accuracy (%)	Validation Accuracy (%)
KNN	99.17	80.84
SVM	98.19	81.5
Random Forest	99.8	77.6
Adaboost	94.89	78.98
XGB	98.46	80
Ensemble Classification	**99.5**	**82.5**

**Table 4:** Results of the patch-level classification of the 4-class classification model.

Class	Precision	Recall	F1 Score	Accuracy (%)
Benign	0.88	0.78	0.82	82.5
Normal	0.75	0.84	0.79	79.5
In situ	0.70	0.87	0.77	79
Invasive	0.91	0.77	0.83	83

**Table 5:** Image-level classification results in terms of precision, recall, F1 score, and accuracy.

Class	Precision	Recall	F1 Score	Accuracy (%)
Benign	1.00	0.96	0.98	97
Normal	0.96	1.00	0.98	98
In situ	0.97	1.00	0.98	97
Invasive	1.00	0.95	0.97	98

**Table 6:** Comparison of the proposed method with some state-of-the-art methods on the ICIAR BACH dataset. Bold font indicates the highest value.

Method	Number of Classes	Patch Wise Accuracy (%)	Image Wise Accuracy (%)
Hela Elmannai et al. [28]	4	_	97.29
Zou et al. [33]	4	_	85.00
Wang et al. [29]	4	_	91.00
Nazeri et al. [30]	4	82.00	95.00
Golatkar A. et al. [31]	4	_	85.00
Golatkar A. et al. [31]	2	_	93.00
Sanyal et al. [32]	4	86.50	95.00
Guo et al. [45]	4	_	87.50
Vang et al. [34]	4	_	87.50
Awan et al. [36]	2	_	87.00
Awan et al. [36]	4	_	83.00
Ferreira et al. [46]	4	_	90.00
Mohamed et al. [35]	4	_	92.5
Proposed method	4	82.50	**97.50**
Proposed method	2	_	**98.60**

## Data Availability

Publicly available datasets were analyzed in this study. The data can be found here: [URL: https://iciar2018-challenge.grand-challenge.org/Dataset/ (accessed on 20 January 2022)].

## Code Availability Statement

The source code of our proposed work can be found at https://github.com/arnablm10/ICIAR2018_BACH_Challenge/tree/main.

## References

[1] Malvia S., Bagadi S.A., Dubey U.S., Saxena S. (2017). Epidemiology of breast cancer in Indian women. Asia-Pac. J. Clin. Oncol..

[2] Siegel R.L., Miller K.D., Jemal A. (2018). Cancer statistics, 2018. CA Cancer J. Clin..

[3] Wang S., Liu Y., Feng Y., Zhang J., Swinnen J., Li Y., Ni Y. (2019). A Review on Curability of Cancers: More Efforts for Novel Therapeutic Options Are Needed. Cancers.

[4] Society A.C. (2019). Breast Cancer Facts & Figures 2019–2020.

[5] Dheeba J., Singh N.A., Selvi S.T. (2014). Computer-aided detection of breast cancer on mammograms: A swarm intelligence optimized wavelet neural network approach. J. Biomed. Inform..

[6] Shen R., Yan K., Tian K., Jiang C., Zhou K. (2019). Breast mass detection from the digitized X-ray mammograms based on the combination of deep active learning and self-paced learning. Futur. Gener. Comput. Syst..

[7] Qi X., Zhang L., Chen Y., Pi Y., Chen Y., Lv Q., Yi Z. (2019). Automated diagnosis of breast ultrasonography images using deep neural networks. Med. Image Anal..

[8] Houssein E.H., Emam M.M., Ali A.A., Suganthan P.N. (2020). Deep and machine learning techniques for medical imaging-based breast cancer: A comprehensive review. Expert Syst. Appl..

[9] Sudharshan P., Petitjean C., Spanhol F., Oliveira L.E., Heutte L., Honeine P. (2019). Multiple instance learning for histopathological breast cancer image classification. Expert Syst. Appl..

[10] Hekler A., Utikal J.S., Enk A.H., Solass W., Schmitt M., Klode J., Schadendorf D., Sondermann W., Franklin C., Bestvater F. (2019). Deep learning outperformed 11 pathologists in the classification of histopathological melanoma images. Eur. J. Cancer.

[11] Comes M.C., Fucci L., Mele F., Bove S., Cristofaro C., De Risi I., Fanizzi A., Milella M., Strippoli S., Zito A. (2022). A deep learning model based on whole slide images to predict disease-free survival in cutaneous melanoma patients. Sci. Rep..

[12] Rajathi G.M. (2020). Optimized radial basis neural network for classification of breast cancer images. J. Ambient. Intell. Humaniz. Comput..

[13] Roy S., Das S., Kar D., Schwenker F., Sarkar R. (2021). Computer Aided Breast Cancer Detection Using Ensembling of Texture and Statistical Image Features. Sensors.

[14] Basavanhally A., Yu E., Xu J., Ganesan S., Feldman M., Tomaszewski J., Madabhushi A. (2011). Incorporating domain knowledge for tubule detection in breast histopathology using O’Callaghan neighborhoods. Medical Imaging 2011: Computer- Aided Diagnosis.

[15] Dundar M.M., Badve S., Bilgin G., Raykar V., Jain R., Sertel O., Gurcan M.N. (2011). Computerized classification of intraductal breast lesions using histopathological images. IEEE Trans. Biomed. Eng..

[16] Melekoodappattu J.G., Dhas A.S., Kandathil B.K., Adarsh K.S. (2022). Breast cancer detection in mammogram: Combining modified CNN and texture feature based approach. J. Ambient. Intell. Humaniz. Comput..

[17] Pramanik P., Mukhopadhyay S., Kaplun D., Sarkar R. (2022). A Deep Feature Selection Method for Tumor Classification in Breast Ultrasound Images. International Conference on Mathematics and Its Applications in New Computer Systems.

[18] Pramanik P., Mukhopadhyay S., Mirjalili S., Sarkar R. (2022). Deep feature selection using local search embedded social ski-driver optimization algorithm for breast cancer detection in mammograms. Neural Comput. Appl..

[19] Majumdar S., Pramanik P., Sarkar R. (2023). Gamma function based ensemble of CNN models for breast cancer detection in histopathology images. Expert Syst. Appl..

[20] Sanyal R., Jethanandani M., Sarkar R. (2020). DAN: Breast Cancer Classification from High-Resolution Histology Images Using Deep Attention Network. Innovations in Computational Intelligence and Computer Vision.

[21] Chattopadhyay S., Dey A., Singh P.K., Oliva D., Cuevas E., Sarkar R. (2022). MTRRE-Net: A deep learning model for detection of breast cancer from histopathological images. Comput. Biol. Med..

[22] Chattopadhyay S., Dey A., Singh P.K., Sarkar R. (2022). DRDA-Net: Dense residual dual-shuffle attention network for breast cancer classification using histopathological images. Comput. Biol. Med..

[23] Bhowal P., Sen S., Velasquez J.D., Sarkar R. (2021). Fuzzy ensemble of deep learning models using choquet fuzzy integral, coalition game and information theory for breast cancer histology classification. Expert Syst. Appl..

[24] Melekoodappattu J.G., Subbian P.S. (2020). Automated breast cancer detection using hybrid extreme learning machine classifier. J. Ambient. Intell. Humaniz. Comput..

[25] Nirmala G., Kumar P.S. (2020). RETRACTED ARTICLE: A novel bat optimized runlength networks (BORN) for an efficient classification of breast cancer. J. Ambient. Intell. Humaniz. Comput..

[26] Kumar D., Batra U., Abraham A., Castillo O., Virmani D. (2021). Breast Cancer Histopathology Image Classification Using Soft Voting Classifier. Proceedings of the 3rd International Conference on Computing Informatics and Networks. Lecture Notes in Networks and Systems.

[27] Preetha R., Jinny S.V. (2020). Retracted Article: Early diagnose breast cancer with PCA-LDA based FER and neuro-fuzzy classification system. J. Ambient. Intell. Humaniz. Comput..

[28] Elmannai H., Hamdi M., AlGarni A. (2021). Deep Learning Models Combining for Breast Cancer Histopathology Image Classification. Int. J. Comput. Intell. Syst..

[29] Wang Y., Sun L., Ma K., Fang J. Breast Cancer Microscope Image Classification Based on CNN with Image Deformation. Proceedings of the International Conference on Image Analysis and Recognition.

[30] Nazeri K., Aminpour A., Ebrahimi M. (2018). Two-Stage Convolu- Tional Neural Network for Breast Cancer Histology Image Classifi- Cation, in International Conference Image Analysis and Recognition.

[31] Golatkar A., Anand D., Sethi A., Campilho A., Karray F., ter Haar Romeny B. (2018). Classification of Breast Cancer Histology Using Deep Learning. Image Analysis and Recognition. ICIAR 2018. Lecture Notes in Computer Science.

[32] Sanyal R., Kar D., Sarkar R. (2021). Carcinoma Type Classification From High-Resolution Breast Microscopy Images Using a Hybrid Ensemble of Deep Convolutional Features and Gradient Boosting Trees Classifiers. IEEE/ACM Trans. Comput. Biol. Bioinform..

[33] Zou Y., Zhang J., Huang S., Liu B. (2021). Breast cancer histopathological image classification using attention high-order deep network. Int. J. Imaging Syst. Technol..

[34] Vang Y.S., Chen Z., Xie X. (2018). Deep Learning Framework for Multi-class Breast Cancer Histology Image Classification. International Conference Image Analysis and Recognition.

[35] Mohamed A., Amer E., Eldin S.N., Khaled J., Hossam M., Elmasry N., Adnan G.T. (2022). The Impact of Data processing and Ensemble on Breast Cancer Detection Using Deep Learning. J. Comput. Commun..

[36] Awan R., Koohbanani N.A., Shaban M., Lisowska A., Rajpoot N. (2018). Context-Aware Learning Using Transferable Features for Classification of Breast Cancer Histology Images. International Conference Image Analysis and Recognition.

[37] Rakhlin A., Shvets A., Iglovikov V.I., Kalinin A.A. (2018). Deep Convolutional Neural Networks for Breast Cancer Histology Image Analysis. International Conference Image Analysis and Recognition.

[38] Aresta G., Araújo T., Kwok S., Chennamsetty S.S., Safwan M., Alex V., Marami B., Prastawa M., Chan M., Donovan M. (2019). BACH: Grand challenge on breast cancer histology images. Med. Image Anal..

[39] Macenko M., Niethammer M., Marron J.S., Borland D., Woosley J.T., Guan X., Schmitt C., Thomas N.E. A method for normalizing histology slides for quantitative analysis. Proceedings of the 2009 IEEE International Symposium on Biomedical Imaging: From Nano to Macro.

[40] Simonyan K., Zisserman A. (2014). Very deep convolutional networks for large-scale image recognition. arXiv.

[41] Szegedy C., Ioffe S., Vanhoucke V., Alemi A.A. Inception-v4, inception-resnet and the impact of residual connections on learning. Proceedings of the Thirty-First AAAI Conference on Artificial Intelligence.

[42] Chollet F. Xception: Deep learning with depthwise separable convolutions. Proceedings of the IEEE Conference on Computer Vision and Pattern Recognition.

[43] He K., Zhang X., Ren S., Sun J. Deep residual learning for image recognition. Proceedings of the IEEE Conference on Computer Vision and Pattern Recognition.

[44] Hands-On Machine Learning with Scikit-Learn, Keras, and TensorFlow by Aurélien Géron. https://www.knowledgeisle.com/wp-content/uploads/2019/12/2-Aur%C3%A9lien-G%C3%A9ron-Hands-On-Machine-Learning-with-Scikit-Learn-Keras-and-Tensorflow_-Concepts-Tools-and-Techniques-to-Build-Intelligent-Systems-O%E2%80%99Reilly-Media-2019.pdf.

[45] Guo Y., Dong H., Song F., Zhu C., Liu J. (2018). Breast Cancer Histology Image Classification Based on Deep Neural Networks. International Conference Image Analysis and Recognition.

[46] Ferreira C.A., Melo T., Sousa P., Meyer M.I., Shakibapour E., Costa P., Campilho A. (2018). Classification of Breast Cancer Histology Images Through Transfer Learning Using a Pre-trained Inception Resnet V2. International Conference Image Analysis and Recognition.

